# The Impact of Sports Involvement on Body Image Perception and Ideals: A Systematic Review and Meta-Analysis

**DOI:** 10.3390/ijerph20065228

**Published:** 2023-03-22

**Authors:** Luciana Zaccagni, Emanuela Gualdi-Russo

**Affiliations:** 1 Department of Neuroscience and Rehabilitation, Faculty of Medicine, Pharmacy and Prevention, University of Ferrara, 44121 Ferrara, Italy; 2 Center for Exercise Science and Sports, University of Ferrara, 44123 Ferrara, Italy

**Keywords:** body dissatisfaction, body image, athletes, sport, body mass index

## Abstract

Concerns about body image may affect athletes, mainly because of specific sports models to achieve successful performance. This systematic review reports on body image dissatisfaction (BID) in athletes following the guidelines for systematic reviews and meta-analyses. From a total of 887 articles identified through a systematic search of electronic databases, 15 articles conducted on 2412 athletes were included in this review. To be eligible for inclusion, the studies must have been published in the last ten years up until September 2022 and analyze body image perception using body figure drawings and anthropometric profiles. The quality of the included studies was evaluated using the adapted Newcastle–Ottawa Scale for observational studies. These studies were compared through thematic analysis of BID to develop four general issues, including gender, sport type and level, and weight status. According to the meta-analyses, the significant medium and small effect sizes found for gender and weight status, respectively, indicated that male athletes had lower BID than females and that, among the latter, normal-weight athletes had a higher BID than underweight ones. The implications and limitations of the included studies are discussed extensively in this review, highlighting the need for further research on BID examined both in the social and the sports contexts. Sports activity should be carried out following healthy lifestyles, and promoting positive BI.

## 1. Introduction

Any form of physical activity can be beneficial to the physical and mental health of youth and adults when undertaken regularly and with sufficient duration and intensity [1,2]. These recommendations are highly relevant since there is simultaneously a worldwide prevalence of physical inactivity and obesity. Indeed, the current sedentary lifestyle is one of the main causes of overweight/obesity [3,4], and this results in a high prevalence of dissatisfaction with the perceived body image (BI) because of the ideal of body thinness prevalent in Western societies [5].

Sports make an important, though underused, contribution to the physical activity of persons of every age [1]. Athletes are engaged in structured and planned physical activity with prominent influences on their physical and mental health. Generally, a positive BI is associated with increased participation in physical activity and sports [6]. BI is considered a multidimensional construct focused on the appearance and function of the body [6]. Body dissatisfaction with an individual’s own physical appearance and body size, as well as discrepancies between actual and ideal dimensions, are cognitive, affective, and perceptual indicators of a negative BI [7]. In essence, a negative or positive BI is shown through the perceptual dimension (how I see myself), and cognitive and affective dimensions (how I think and feel about my physical appearance) [7].

In a sports context, a more favorable BI would depend on actual physical changes resulting from the sport practiced (e.g., body shape), perceived changes in the physique, and building self-efficacy and confidence. However, this relationship is by no means simple: while physical activity practice contributes to raising self-confidence through a number of discernible physical changes (e.g., an increase in fat-free-mass) resulting in improved BI satisfaction, BI may, in turn, induce motivation or dissuasion for physical activity and sports participation [6]. Thus, for example, exercise addiction arises from a misperception of BI [8] and can also result in decreased performance owing to overload and physical burnout [9].

An important aspect to consider is whether dissatisfaction is influenced by the type of exercise practiced. Some differences in body dissatisfaction recorded among practitioners of different sports [10] might depend on the importance of body weight and body thinness within that sport [11]. A particular relevance of physical appearance can be found in aesthetic sports, such as rhythmic gymnastics. In this case, the assessment of the athlete considers his/her morpho-kinetic abilities based on well-coded aesthetic requirements. Therefore, in addition to performance, the athlete’s physical appearance strongly contributes to the judgment, so much so that a prevalence of dissatisfaction among athletes involved in aesthetic sports has been reported in several studies [12,13]. With particular reference to the female gender, a higher risk of body concerns was observed in gymnastics than in swimming and long-distance running [14]. However, in these cases, it is important to distinguish the “sport” body image dissatisfaction (sport-BID = perceived discrepancy between current and ideal body size for sport) from the general body image dissatisfaction (BID) [15,16]. Indeed, the literature [15] shows that athletes, especially in aesthetic sports, would not be driven toward dieting and pathological weight control because of general BID, but because of the specific needs of the sport they play. Greenleaf [17] distinguished the BI of the athlete within an athletic context from a social BI that relates to the context of everyday life. Satisfaction/dissatisfaction with one’s body image will therefore depend not only on one’s physical appearance, but also on the social or sports environment of reference. Although athletes tend to be more satisfied than non-athletes in the social environment [18], in the sports environment, athletes often are under pressure from coaches and athletic trainers to achieve and retain a body that is favorable to their respective sport [19]. Regarding aesthetic sports, for example, it has been found that the ideal sports figure of the female gymnast does not coincide with the ideal figure in everyday life, being leaner [16].

According to previous literature reviews, athletes have a more positive BI than non-athletes both considering studies published between 1975 and 2000 [18] and between 2000 and 2012 [14]. Although both reviews made this comparison taking into account age and competitive level, no gender comparisons were made in either the first review (which reports a small percentage of males) or the second review (focused exclusively on females). The majority of studies in this field concern eating disorders, showing a higher incidence of disordered eating in athletes, particularly in aesthetic and weight-class-dependent sports [20]. De Bruin et al. [15] analyzed the role of BI in athletes’ disordered eating, showing that the athletic BI contributes greatly to this symptomatology. To date, research has focused primarily on the impact of negative BI on the athlete or pathological aspects without thoroughly considering the framework [21]. In other words, we believe that the extent to which the type and level of sport played and the athlete’s characteristics (body composition, age, gender, and ethnicity) affects the athlete’s BID has not been sufficiently investigated. As well, little attention has so far been paid to general BID compared to that of the sports context (sport-BID).

The main purpose of this systematic review and meta-analysis was to assess and understand the level of satisfaction/dissatisfaction with BI in athletes practicing different types of sports at various levels. The secondary aim was to summarize the empirical findings from the most recent studies, focusing on the possible effects of sports’ impact on body perception and ideals. In particular, we analyzed the impact of the following factors on the BI perception and BID (acronym used for the general BID in this text) or sport-BID: gender, sport type, sport level, and individual anthropometric characteristics (at least BMI). Finally, in this systematic review, we have attempted to report the current limitations of the scholarship in this field and to formulate suggestions for future research and improvement in the sports field.

## 2. Materials and Methods

### 2.1. Search Strategy and Eligibility Criteria

We conducted a systematic literature review following the PRISMA guidelines for systematic reviews and meta-analyses (PRISMA) [22]. The PRISMA checklist is provided in the Supplementary Materials (Table S1). The protocol of this review registered in PROSPERO (International Prospective Register of Systematic Reviews) can be found through the following registration number: CRD42022381243.

Searches for articles published over the past 10 years (up to 12 September 2022) were carried out in the following two databases: PubMed and Web of Science. The following combination of search terms was used: (“body image” OR “body dissatisfaction” OR “body satisfaction” OR “body representation” OR “silhouette scale” OR “ideal body image”) AND (anthropometry OR BMI OR weight OR “body composition”) AND (“sport activity” OR sport* OR athlete). The PubMed filters used to narrow the search results were publication date, and age ticking the adolescent (13–18 years) and adult (19–44 years) categories.

Both co-authors independently manually reviewed the potential articles for eligibility, first based on the title and abstract, and then the full text of the article. Any disputes about eligibility were resolved by further analysis and discussion between the co-authors. Finally, the reference lists of the selected articles were reviewed to search for additional articles not previously collected.

The inclusion criteria for considering an article eligible were as follows: (1) measure of body image by body silhouettes scale; (2) participation in sports activity at competitive level; (3) data on anthropometric measurements; (4) full-text availability; (5) available in English; (6) participants selected from non-clinical populations. Regarding participants, we excluded studies that considered an age range other than the one used (13–44 years). Concerning subject matter, we excluded studies conducted to develop or validate new BI screening tools, or those that considered dissatisfaction with specific body features or body parts (apart from muscularity, which is more closely related to sports activity). Concerning the tool of the BI measure, we excluded studies that only considered an assessment with questionnaires to reduce the heterogeneity of the BI measure. Studies conducted on the same sample were not included, except when supplementary information on a subsample was provided [23]. Finally, we did not include literature reviews, books (or book chapters), editorials and commentaries, case studies, protocol studies, or conference proceedings.

We extracted, when possible, the following information from each study: authors’ name and year of publication, study design, sample characteristics (number, gender, age, country), anthropometric characteristics of participants, type and level of sport played, BI measure, and BID.

The retrieved data were summarized qualitatively and shown in table format, reporting the studies in alphabetical order.

### 2.2. Data Analysis

The meta-analyses were performed using the MedCalc Statistical Software (MedCalc Software Ltd., Ostend, Belgium). The effect sizes were calculated via the means, standard deviations, and sample sizes of studies that reported these parameters for BID or sport-BID according to gender, type and level of sport, or weight status. In particular, the standardized mean difference (SMD) with 95%CI (confidence interval) and relative weight of each study, and the total SMD were calculated and tabulated. The findings of the meta-analysis were visually displayed by forest plots. The SMD was interpreted using Cohen’s cut-offs [24] as small (0 to 0.2), medium (0.3 to 0.7), and large (≥0.8). If the CI does not include zero, then the effect size is statistically significant at the *p* < 0.05 level. 

We considered the heterogeneity test (Cochran’s Q) to measure the variation in the study results among the studies in the meta-analysis. In particular, we chose the random-effects model because it is the most appropriate to use in social science research as it assumes variability in the effect sizes across the included studies, according to the indications of Borenstein et al. [25]. As the test is susceptible to the number of studies included in the meta-analysis, we also considered the I^2^ value, which gives a better measure of the consistency between studies in a meta-analysis. A value of 0% indicates no observed heterogeneity, while larger values show increasing heterogeneity; based on existing recommendations, I^2^ values of 25, 50, and 75% were considered low, moderate, and high, respectively [26]. 

### 2.3. Risk of Bias

Following PRISMA recommendations for systematic reviews [22], the two co-authors independently assessed the risk of bias of included studies using the adapted Newcastle–Ottawa Scale (NOS) for observational studies [27]. The NOS evaluation criteria include (1) clearness of the objective of the study; (2) selection of the sample (its representativeness and size; response rate; ascertainment of exposure); (3) comparability (checking for confounding factors; comparability of participants from various outcome groups); and (4) outcome (evaluation; statistical tests). Assessment of exposure, as required in criterion 2, was performed with regard to the figural scale employed (validated or non-validated). The overall score for each study (ranging from 0 to 16) was calculated from the individual component ratings reported. Following Hillen et al. [27], we regarded studies that had received a score of 13–16 out of a possible 16 points (scores > 75%) as of low risk of bias, studies with a score of 9–12 points (scores > 50%) as of moderate risk, and studies of high risk of bias for scores ≤ 8 points (scores ≤ 50%). A low overall score denotes an increased risk of study bias. 

## 3. Results

### 3.1. Selection of Studies

**Figure 1:**
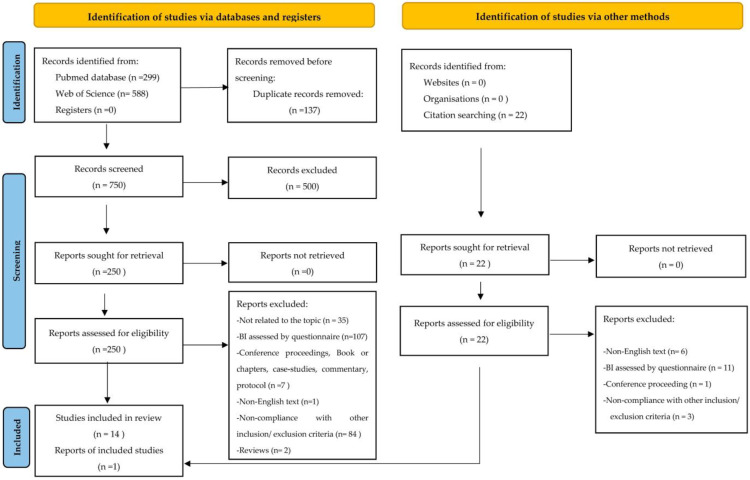
Flow chart of the study selection process (PRISMA 2020 flow diagram) [22].

A total of 887 articles were found on PubMed and Web of Science. Of these, 137 were duplicates, resulting in 750 articles to be verified for eligibility. After reading the titles and abstracts, 500 studies were excluded, and 250 studies were read in full. Fourteen studies were included in this review because they met the eligibility criteria, and after reading their references, one additional study was included, resulting in fifteen articles. The article selection process is presented in Figure 1.

### 3.2. Characteristics of Studies

The review included 15 studies on a total of 2412 athletes (Table 1).

All of the studies except a longitudinal one had a cross-sectional design. Seven studies were carried out in America (four in Brazil and three in the USA), six in Europe (two in Portugal, two in Italy, one each in Germany and Spain), one in Oceania (in Australia), and one in Asia (in Turkey). The number of subjects surveyed in the studies ranged from a minimum of 10 to a maximum of 725: 6 studies had up to 100 subjects, 5 studies had up to 200, and 4 had a sample size above 200. The sample size was usually inversely related to the level of sports performance. Six studies considered adolescent subjects (13–18 years) and the remaining nine adults; six studies considered only the female gender, four only the male gender, and five both genders. The youngest sample was the 14-year-old boys and girls (mean age: 14.0 ± 2.3 years) practicing aesthetic sports from elite sports schools and Olympic training centers in Germany in the longitudinal study of Krentz and Warschburger [41], and the oldest sample was that of the Italian male bodybuilders (mean age: 33 ± 7 years) from Santarnecchi and Dettore [45]. Most of the studies (12 out 15) concerned athletes engaged in aesthetic sports (dancers, rhythmic gymnasts, figure skaters, cheerleaders), two studies concerned male bodybuilders (Turkish and Italian bodybuilders), and one female soccer player (Spanish amateur non-elite soccer players). 

As for the anthropometric profile, most of the studies reported BMI calculated from self-reported weight and height; only in four studies were height and weight measured objectively and, in two of these, the percentage of fat mass was also assessed by DEXA [34] or by the skinfold method [31]. Finally, Devrim et al. [32], in their study, asked bodybuilders to self-report height, weight, body fat percentage, and fat-free mass index. Based on the mean BMI values, the athletes resulted in normal weight status: generally, aesthetic sports athletes had a lower BMI than non-aesthetic ones, often used as a control group. Considering the level of sport, elite aesthetic athletes had a lower BMI than non-elites, and the opposite was found in bodybuilding, where the BMI increased with the level of performance.

The studies included in the review assessed the BI perception using a body silhouette scale. In five studies on adolescent athletes, the authors used the Contour Drawing Rating Scale proposed by Thompson and Gray [37], while Borrione et al. [28], in their study on adolescent Italian and international rhythmic gymnasts, used the Figure Rating Scale developed by Stunkard et al. [29]. In adult athletes, the BI perception was assessed using Stunkard et al.’s silhouettes [29] (four studies), Pinto et al. [42] used the Muscle Silhouette Measure developed by Frederick et al. [43,44], de Medeiros Eufrásio et al. [34] used the Kakeshita et al. silhouettes [35], and Godoy-Izquierdo et al. [38] used the silhouettes proposed by Ramirez et al. [39]. Two studies on male bodybuilders used the Bodybuilder Image Grid (BIG), 30 silhouettes of male figures that are used to measure body image perceptions of male athletes, especially for bodybuilders [33]. The reliability and validity of all of the silhouette scales used in the included studies were assessed [33,35,43,50,51]. Three studies reported the percentage of people satisfied and dissatisfied with their body image; the others reported the ideal and current figures and their difference as a measure of dissatisfaction (BID). In particular, the athletes engaged in sports focused on leanness and thin shape showed negative BID values or a high percentage of dissatisfaction with being overweight. Conversely, female soccer players [38], male elite or non-elite artistic gymnasts [23,42], and male elite dancers [22] showed positive values of the BID to better meet the physical demands of the sport practiced. The bodybuilders studied by Devrim et al. [32] and Santarnecchi et al. [45] wanted to be leaner, but more muscular than they were. Five studies [23,40,41,48,49] on athletes engaged in sports focused on leanness reported the sport-BID, which measures dissatisfaction using the difference between the ideal silhouette for the sport practiced and the current silhouette of the athlete. All of the athletes except the male adolescent dancers and male non-elite gymnasts of Francisco et al. [23] reported negative values of the sport-BID.

### 3.3. Meta-Analyses

#### 3.3.1. BID by Gender

Four studies (27%) included in the review analyzed athletes of both genders, but Krentz and Warschburger [41], Cardoso et al. [30], and Da Silva et al. [31] did not consider BID, so only the study (7%) of Francisco et al. [36] reported the necessary data to calculate the effect size. They reported the number, mean, and SD of male and female elite aesthetic athletes, non-elite aesthetic athletes, and non-aesthetic athletes (Table 2).

The meta-analysis on a total of 725 athletes revealed a medium and significant effect of gender on BID, with girls more dissatisfied than boys (SMD = −0.56, *p* < 0.001). The Cochran’s Q test and I^2^ statistics revealed no heterogeneity among the studies (χ^2^ = 0.558, DF = 2, *p* = 0.757; I^2^ = 0.0%).

#### 3.3.2. BID by Type of Sports: Aesthetic Sports vs. Non-Aesthetic Sports

Only three studies (20%) on female athletes (two on adolescents [28,36] and one on young adults [40]) reported the necessary data to perform a meta-analysis on the effect of sport type on BID based on a total of 582 female subjects: 201 girls engaged in aesthetic sports (at elite level) and 381 girls engaged in non-aesthetic sports (Table 3).

Meta-analysis revealed a non-significant effect of the type of sports on BID in female athletes (SMD = −0.02; *p* = 0.899). The Cochran’s Q test and I^2^ statistics revealed a significant heterogeneity among the studies considered (χ^2^ = 7.68, DF = 2, *p* = 0.02; I^2^ = 73.96%).

#### 3.3.3. BID by the Level of Sport: Elite Level vs. Non-Elite Level in Aesthetic Sports

The same studies examined in the above analysis were used to perform a meta-analysis on the effect of the level of aesthetic sports on BID (Table 4) based on 420 female subjects.

The meta-analysis revealed a small and non-significant effect of the elite level in aesthetic sports on BID compared to the non-elite level (SMD = −0.14; *p* = 0.293). The Cochran’s Q test (χ^2^ = 3.334, DF = 2, *p* = 0.189) and I^2^ statistics (40.01%) revealed a moderate and non-significant heterogeneity among the studies considered.

#### 3.3.4. BID by Weight Status: Underweight Athletes vs. Normal-Weight Athletes

Taking into account the mean BMI values, only the study of Borrione et al. [28] considered underweight and normal-weight athletes: the international- and national-level rhythmic gymnasts’ mean BMI value fell in the underweight category, while the controls’ (athletes practicing basketball, volleyball, Taekwondo) mean BMI values fell into the normal-weight category. The meta-analysis revealed a small (SMD = 0.35), but significant (*p* = 0.014) effect of the weight status on BID, with underweight athletes less dissatisfied than those of normal weight, despite the fact that the former practiced an aesthetic sport and the latter sports non-focused on leanness. The Cochran’s Q test (χ^2^ = 0.355, DF = 1, *p* = 0.551) and I^2^ statistics (0%) revealed no heterogeneity among the samples considered (Table 5).

#### 3.3.5. Sport-BID by Gender

Four studies (27%) provided information about the influence of gender on sport-BID; a meta-analysis was performed based on a total of 501 participants (413 females and 88 males), all engaged in aesthetic sports at an elite level. Voelker et al. [48] reported data for female figure skaters and Voelker et al. [49] reported data for male figure skaters. The meta-analysis revealed a moderate and significant effect of gender on sport-BID: female athletes are more dissatisfied about their actual body size concerning their ideal sport-practiced body size compared to the males (SMD = −0.74; *p* < 0.001). The Cochran’s Q test (χ^2^ = 3.935, DF = 3, *p* = 0.269) and I^2^ statistics (23.76%) revealed no heterogeneity among the studies considered (Table 6).

### 3.4. Risk of Bias

The NOS scores ranged from 6 to 14 (from 37.5% to 87.5%): the majority of the studies (10 equal to 66.7%) had a moderate risk of bias, and the remaining 5 studies were of low (2 equal to 13%) or high (3 equal to 20%) risk (Table 1 and Table S2). All of the studies clearly stated the aims and used validated measurement tools for BI assessment considered in the ascertainment of the exposure. Twelve studies (80%) provided an adequate description of the sampling procedure, although only three (20%) were carried out on a random sample and three studies reported a response rate. Eight studies (53.3%) controlled for potential confounding factors and twelve studies (80%) clearly described the statistical tests used.

## 4. Discussion

This study provides an updated literature review for the past ten years regarding BI in adolescent and adult athletes, giving some insight into the variables that could affect the BID. Previous reviews have mainly focused on the perception of BI in the female gender [52], whereas this review aims to potentially verify this pattern in the male gender as well. Taking into account the serious negative effects of BI disturbance, we conducted the present literature review focusing mainly on BI perception, and general and sport dissatisfactions, obtained as the discrepancy between the actual and general or sport ideal figure on the silhouettes scale, and assessing the influence of gender, type of sport played, level of performance, and weight status.

Based on the 15 studies reviewed, we found a greater BID in female athletes than in male athletes. It is well known from the literature that females are generally more dissatisfied than males and are more likely to prefer thinner silhouettes than men [5,53,54]. Although the importance of sports participation in improving the perception and acceptance of one’s body image has been demonstrated [55], it is not surprising that even among athletes, these gender disparities can be observed [7,56]. From childhood, the female gender seems to be more aware of the effect of body weight on BI than males, resulting in greater dissatisfaction with their appearance than their male peers [57,58]. This different pattern may depend on the greater importance placed by females on aesthetic aspects and the lower importance they place on functional bodily aspects compared to males [59].

Compared with the non-sports population, athletes are generally believed to be more satisfied because their physique better reflects the ideal characteristics of the Western world (thinness in the female gender, muscularity in the male gender) [18]. However, comparisons between athletes and non-sports individuals were generally absent in the revised articles, except for the study by de Medeiros Eufrásio et al. [34], which confirms a lower dissatisfaction with BI in athletes than in sedentary people. In general, a positive influence of physical activity on psychological characteristics (such as self-esteem) related to positive BI has been reported in the literature [55,60]. However, this pattern could depend on the type of sport practiced, reaching higher values of dissatisfaction in the athletes of aesthetic/lean sports than non-aesthetic/non-lean sports [61,62]. In other words, the BID seems to be higher in individuals who participate in weight-sensitive sports, such as aesthetic (e.g., gymnastics), weight class (e.g., boxing), gravitational endurance (e.g., long-distance running), and gravitational technical (e.g., high jump) sports, because weight has a significant influence on performance [63]. In the present systematic review, sports were found to affect BID differently depending on the sport type. In particular, ballet dancers [23,30,34] appeared to be more dissatisfied than athletes in other specialties. Their dissatisfaction was predominantly related to the perception of being overweight [30] with a greater desire for thinness in female dancers than in male dancers [30,31]. These results confirm the particular attitude of dancers toward body dissatisfaction reported in the literature and the possible subsequent risk of eating disorders such as anorexia and bulimia [64,65]. Although the sports considered in this review were mainly aesthetic, we could verify a different body IBI between athletes practicing some sports such as gymnastics, dancing, skating, and cheerleading compared to bodybuilding and soccer. While the athletes’ dissatisfaction in the first group of sports leads to a desire for a leaner and more slender body, especially in elite female rhythmic gymnasts, in the second group of sports, the athlete’s wish is to have a leaner and more muscular shape, especially in bodybuilding [32]. Another important aspect to consider seems to be the sport level, as higher body satisfaction in high-level athletes has been pointed out in the literature [62,66]. However, this systematic review only indicates a partial confirmation of this trend ([28,40] for athletes in non-leanness sports; [35] for male athletes), while other studies show the opposite trend ([35] for elite female athletes; [40] for elite athletes of leanness sports). Most athletes (especially females) reported pressure from coaches regarding body shape [40,41] to better align with the ideals and norms of that sport, which may result in greater risks of BID and disordered eating behaviors following the literature [67,68].

The literature shows different phenotypes of athletes concerning anthropometric characteristics and body composition (% fat, FM, FFM) related to sport, gender, and competitive level [69,70]. In sports such as rhythmic and artistic gymnastics and figure skating, athletes believe they can achieve higher scores when their body mass and shape conform to a specific body ideal for that given sport [71]. However, only three of the included studies reported body composition parameters [31,32,34], unlike the BMI reported in all 15 studies. In general, a definite association is known between body weight perception and BI, with significant positive correlations between BMI and BID/restrictive eating [72]. More specifically, lower BMI values were found in aesthetic sports, especially in elite athletes. Therefore, estimates of total body composition, which would be essential for assessing functional mass contributing to strength and power production and thus to performance [70], are lacking, therefore preventing us from considering these important characteristics in this review.

Another important aspect to consider concerns the possible differences between the general BID and sport-specific dissatisfaction that is influenced by the ideal shape and size for that sport [15], especially in aesthetic sports [73]. Gymnasts, for example, seem confident that “thin is going to win” rather than “thin is beautiful” [74]. Since the perfect body for the best performance in a specific sport does not always match the body ideal in society [75], a misunderstanding in the assessment and interpretation of an athlete’s BID can result when BI in the athletic context is not separately recorded compared to general BI. Although the athlete’s perception of the body may depend on the context in which he or she is [49], only three studies among those reviewed collected BI data separately to obtain both BID and sport-BID [23,40,48]. Two other studies among those included examined the sport-BID alone [41,49] and the remaining studies only considered the general BID. Two of the three studies that evaluated the two different BID types showed higher sport-BID values than the general BID values [23,48], highlighting the athlete’s awareness of having a body figure that fits society’s ideals, but that the ideals for achieving success in sports are more stringent.

Following the systematic review, we tried to obtain a quantitatively pooled estimation from a selected number of studies using meta-analysis. This statistical summary may allow us to reach meta-analytic conclusions on general or sport-BID in athletes by comparing standardized effect sizes across studies according to specific variables (gender; type or level of sport; weight status).

*Gender*—Based on four samples of athletes of both genders practicing aesthetic sports, we found significant gender differences in general and sport-BID, with female athletes more dissatisfied than male athletes (overall effect size: medium), especially in the case of sport-BID in elite dancers (large effect size). These gender differences are not surprising, as dissatisfaction with BI is a constant in all-female populations, as mentioned above. The effects on sport-BID were found to have low heterogeneity and those on general BID were strongly homogeneous. In the latter case, the degree of heterogeneity was calculated from different subsamples in the same study [23], unlike the degree of heterogeneity on sport-BID, which was based on four samples from four different studies [36,48,49].

*Sport type and level*—Meta-analyses based on three specific available studies showed a non-significant small effect size, indicating that sport type (aesthetic vs. non-aesthetic sports) and level (elite vs. non-elite level) led to changes in BID. In this case, the non-significance could also depend on low power because of the small number of studies considered. Greater BID was found in aesthetic and elite-level sports in two of three studies. The effect size value obtained falls within the range of the small-to-medium effect already found in some previous reviews on the effect of physical activity on BI [52,76]. Moreover, given the heterogeneity of studies ranging from moderate (competitive level) to substantial (sport type), we sought a plausible interpretation. Examining the three studies, we found that one sample was examined in Portugal [36], the other in Australia [40], and the last [28] is mixed in terms of nationality, including Italian gymnasts and gymnasts from the World Rhythmic Gymnastics Championship. Consequently, ethnic differences between the samples are plausible.

*Weight status*—Based on two subsamples of underweight and normal-weight female athletes from the study of Borrione et al. [28], a small significant effect size of weight status on general BID was found, with lower BID in underweight than in normal-weight athletes. The homogeneity between the subsamples is demonstrated by the large overlap between the confidence intervals in the forest plot and by the heterogeneity tests.

Regarding the risk of bias in the studies reviewed, caution should be taken in evaluating the findings, as one-fifth of them are high-risk. The participating athletes were generally not randomly selected and sometimes the sample size was very small (even just 10 athletes) and lacked a control group. The body composition characteristics of athletes have not generally been reported, forcing us to refer only to the BMI, despite the known weaknesses of this index [16,77]. Additionally, in the majority of the studies, BMI was based on uncertain stature and weight data, since these anthropometric measurements were reported by the participants. Moreover, although ethnicity is considered an important variable [76], we could not assess the influence of athletes’ ethnicity on BID due to the general lack of information in the studies reviewed. Lastly, all but one of the studies we included in this review were cross-sectional, thus not providing the same level of evidence as longitudinal studies.

This systematic review and meta-analysis offered a literature overview of BID in athletes over the past decade, identifying possible literature shortcomings to enhance upcoming research. The strengths of this review include adherence to the PRISMA statement and the inclusion of meta-analyses. To limit the heterogeneity of the methods for assessing BI perception across studies, a specific strength was the exclusive inclusion of studies that assessed BI through figure scales. Despite these strengths, a few limitations should be mentioned. The decision to conduct a literature review that only refers to the last 10 years can be seen as a limitation, even though this has made it possible to follow a criterion of greater homogeneity in body ideals while avoiding possible trends over time with changes in ideal beauty. In this regard, the impact that the recent widespread use of social media seems to have on body ideals should be emphasized [78]. Two clear limitations are the review of articles in English only and the search for articles limited to only two major databases (PubMed and Web of Science). Finally, due to the heterogeneity of the methodologies used in different research, a small number of studies were used in the meta-analyses, causing possible limitations in the interpretation of the results. More generally, further limitations depend on the limitations of the studies reviewed, as previously pointed out.

Future research using more robust study designs with a clear differentiation between general and sport BID will allow progress in a better understanding of the BI perception and body ideals in the athlete by analyzing mediating and moderating variables.

## 5. Conclusions

Body image is an important component of athletes’ health. Poor body image can lead to negative consequences, including eating disorders. The descriptive data in this systematic review and the small size for the effects of sport type and level on body image observed with the meta-analysis suggest that a consistent BID represents a general characteristic of the athlete in aesthetic sports. Female athletes at a high level especially tend to perceive their bodies to be not quite adequate in terms of the model needed to achieve success in sports, and are generally unsatisfied with their BI in sports, but not in the social context in which they live.

We strongly suggest promoting programs aimed at lowering the risk of disordered eating, such as nutritional counseling and sports counseling, following the recommendations of the World Health Organization, fostering the development of self-esteem and positive BI.

## Figures and Tables

**Table 1:** Summary of selected studies included in the systematic review: sports information, anthropometric profile, and body image assessment of the samples.

Author (Year) -Study Design-	Athlete Population *Sport, Level*	Participant Characteristics *Gender, N*	Age (Years) *M ± SD*	Anthropometric Measures, BMI (kg/m^2^), and Body Composition	Measure of BI	Main Findings	Study Quality
Borrione et al. (2013) [28] -cross-sectional-	Elite rhythmic gymnasts	161 females 81 gymnasts: 20 international (Int G), 61 Italian (It G); 80 controls (C) (practicing basketball, volleyball, Taekwondo, fitness)	Int G: 18.1 ± 2.4 It G: 16.0 ± 2.9 C: 17.1 ± 3.2	BMI Int G: 17.4 ± 1.4 It G: 16.8 ± 1.8 C: 21.1 ± 2.2	According to Stunkard et al. [29] Int G: CBI; IBI; BID 2.5 ± 0.7; 2.1 ± 0.5; −0.5 ± 0.8 It G: CBI; IBI; BID 3.2 ± 0.9; 2.4 ± 0.7; −0.7 ± 0.9 C: CBI; IBI; BID 4.7 ± 1.5; 3.7 ± 1.1; −1.0 ± 1.1	Overall sample presented dissatisfaction with BI (wanted to be more slender); elite gymnasts had the right perception of their body; lower-level gymnasts and controls perceived themselves as fatter than the real size	11 moderate
Cardoso et al. (2021) [30] -cross-sectional-	Brazilian professional ballroom dancers with 6–10 years of professional experience in ballroom dancing. They practiced ballroom dancing from 5 to 7 times per week	133 females 187 males;	31.48 ± 8.63	BMI (self-reported data): M + F: 24.42 ± 4.02 F: 22.84 ± 4.26 M: 25.49 ± 3.48	According to Stunkard et al. [29] F 34.6% satisfied 56.4% dissatisfied with OW 9.0% dissatisfied with thinness M 19.3% satisfied 55.5% dissatisfied with OW 25.1% dissatisfied with thinness	The majority of the dancers of both genders were dissatisfied with their BI. Age and BMI were the variables that most influenced their dissatisfaction	12 moderate
Da Silva et al. (2016) [31] -cross-sectional-	Professional ballet dancers in Sao Paulo, Brazil. Level not specified	5 women 5 men	Range: 20–41 F: 26.8 ± 8.8 M: 29.6 ± 8.6.	F: W: 55.5 ± 2.3 kg BMI: 20.7 ± 0.4 kg/m^2^ Waist: 74.1 ± 3.0 cm %F: 17.3 ± 2.0 M: W: 75 ± 9 kg BMI: 23.5 ± 2.6 kg/m^2^ Waist: 82.7 ± 8.0 cm %F: 9.6 ± 2.5 (Waist measured at umbilical scar)	According to Stunkard et al. [29] % of silhouettes self-perceived as ideal vs actual: F: 40% equal; 60% lesser. M: 20% equal; 40% lesser; 40% bigger	The Brazilian dancers were eutrophic but women showed higher adiposity and a greater desire to be thinner than men	6 low
Devrim et al. (2018) [32] -cross-sectional-	Competitive and non-competitive bodybuilders from 4 bodybuilding gym centers in Ankara, Turkey	120 male bodybuilders divided into 2 groups: 62 Competitive (C) (practicing bodybuilding 362.0 ± 128.46 min/week) and 58 Non-Competitive (NC) (practicing bodybuilding 342.6 ± 130.20 min/week)	Total: 28.25 ± 9.17 C: 31.05 ± 10.60 NC: 25.63 ± 6.67	(self-reported data) Total W: 83.72 ± 12.97 kg H: 178.91 ± 6.36 cm %F: 13.57 ± 4.00 FFMI: 22.58 ± 2.91 kg/m^2^ C FFMI: 24.09 ± 3.05 kg/m^2^ NC FFMI: 21.18 ± 1.93 kg/m^2^ General relationship between FFMI and %F r = 0.049 (non-significant)	According to Bodybuilder Image Grid Original (BIG O) and Scaled (BIG S) Hildebrandt et al. [33] BIG O Fat mass scores Current: 47.06 ± 22.32 Ideal: 28.96 ± 19.61 Muscle mass scores Current: 54.31 ± 20.09 Ideal: 72.24 ± 14.63 No difference between C and NC BIG S Current: column 3 (%F 16.5) Ideal: column 2 (%F 10)	Men who suffer from eating disorders wish to have a more muscular shape, particularly in sports related to body weight, such as bodybuilding. Bodybuilders want to be leaner and more muscular than they are	10 moderate
de Medeiros Eufrásio et al. (2021) [34] -cross-sectional-	Brazilian amateur female adult dancers who were training in classical ballet at an intermediate/advanced level (training at least 6 h per week and using point shoes for at least 1 year)	57 females (N = 19 ballet dancers, N = 19 gym users (GU), N = 19 sedentary (SED))	Median age 24.0 years for Ballet dancers, 25.0 years for GU and SED	BMI: Ballet dancers: 20.9 ± 2.4 GU: 22.1 ± 2.4 SED: 23.2 ± 3.5 %Fat Median (Q1–Q3); by Dexa Ballet dancers: 31.2 (21.6–34.4) GU: 34.3 (30.2–38.3) SED: 38.9 (34.2–42.2)	According to Kakeshita et al. [35] 75% of Ballet dancers were dissatisfied with their BI (91.7% wanted to be smaller and 8.3% wanted to be bigger) 70.6% of GU and 100% of SED were dissatisfied	BID was significantly lower in the ballet dancers compared to the sedentary women. As ballet dancers practiced classical ballet for many years and for many hours/week, this practice was associated with a thinner body, putting the ballet dancers closer to the shape idealized by most women	11 moderate
Francisco et al. (2013) [36] -cross-sectional-	Elite athletes who are internationally competitive gymnasts and professional dance students and non-elite athletes who are gymnasts from lower levels of competition and recreational dancers in Portugal	725 adolescents (453 F, 272 M). They were divided into 2 groups: 245 aesthetic athletes (54.3% elite) and 480 controls (they did no aesthetic sports).	Total 15.34 ± 2.12	BMI (self-reported data) *F:* Elite (N = 101): 19.16 ± 2.15 Non-elite (N = 99): 19.52 ± 2.55 Control (N = 253): 20.36 ± 2.88 *M* Elite (N = 30): 20.43 ± 2.71 Non-elite (N = 15): 21.43 ± 3.29 Control (N = 227): 21.10 ± 3.30	According to Thompson and Gray [37] BID F: Elite: −0.88 ± 1.21 Non-Elite: −0.51 ± 1.19 Control: −0.81 ± 1.31 M: Elite: −0.11 ± 0.83 Non-Elite: 0.27 ± 0.96 Control: −0.17 ± 1.09	Elite female athletes displayed a higher risk of the development of eating disorders than non-elite athletes and controls. BID is predicted by the same risk factors (gender, BMI, social pressure) in all 3 groups, and is the strongest predictor of eating disorders in elite athletes, but not in non-elite athletes or controls	12 moderate
Francisco et al. (2012) [23] -cross-sectional-	Aesthetic performers: ballet dancers and gymnasts of 4 disciplines: acrobatics, trampoline, rhythmic gymnasts, and artistic gymnasts.	113 ballet dancers (88.5% F) 136 gymnasts (75%F) According to level: 66 elite dancers (53 F + 13 M); 47 F non-elite dancers; 69 elite gymnasts (international competitions) (50 F + 19 M) 67 non-elite gymnasts (52 F + 15 M)	Elite dancers: 14.53 ± 2.28 Non-elite dancers: 14.57 ± 2.30 Elite gymnasts: 16.33 ± 2.59 Non-elite gymnasts: 15.27 ± 2.56	Dancers elite F (N = 53): 18.12 ± 1.85 non-elite F (N = 47): 19.04 ± 2.26 elite M (N = 13): 18.22 ± 1.68 Gymnasts elite F (N = 50): 20.16 ± 1.99 non-elite F (N = 52): 19.94 ± 2.26 elite M (N = 19): 21.83 ± 2.26 non-elite M (N = 15): 21.43 ± 3.29	BID Dancers Elite F: −0.89 ± 1.25 Non-elite F: −0.23 ± 0.94 Elite M: 0.31 ± 0.86 Gymnasts Elite F: −0.84 ± 1.30 Non-elite F: −0.75 ± 1.34 Elite M: −0.26 ± 0.81 Non-elite M: 0.27 ± 0.96 Sport-BID Dancers Elite F: −1.45 ± 1.32 Non-elite F −0.72 ± 0.95 Elite M: 0.08 ± 0.95 Gymnasts Elite F: −1.20 ± 1.04 Non-elite F: −1.00 ± 1.02 Elite M: −0.58 ± 1.02 Non-elite M: 0.07 ± 1.03	Dissatisfaction with BI specific to the practice of an aesthetic activity is the best predictor of eating disorders, compared to dissatisfaction with body image in general, especially in dancers	12 moderate
Godoy-Izquierdo and Diaz (2021) [38] -cross-sectional-	Spanish amateur non-elite female soccer players	45 females	20.9 ± 7.5 (range: 13–44)	(self-reported data) W: 62.7 ± 13.7 (39–104 range) kg BMI: 23.1 ± 3.9 Weight Status 11.1% UW 60% NW 22.2% OW 6.7% Obese	According to Ramirez et al. [39] CBI: 7.7 ± 2.2 IBI: 9.8 ± 1.6 BID: 2.0 ± 1.8	The soccer players showed self-representation of their bodies that corresponded to their reality as athletes, but their body ideals were also more demanding in terms of low fat and high muscularity, in association with the functionality of their body and their athletic activity to adjust more to the ideal body determined by the physical demands of soccer	10 moderate
Kong and Harris (2015) [40] -cross-sectional-	Elite (E), Recreational (R), and Non-competitive (NC) female athletes competing in leanness-focused sports and non-leanness-focused sports in Australia. Training (*h*/*w*) E:17.28 ± 9.22 R: 7.26 ± 3.81 NC: 5.63 ± 4.25	320 F divided into groups: *Leanness sports:* 80 E, 59 R, 35 NC *Non-Leanness sports:* 48 E, 53 R, 45 NC.	Total 21.7 ± 3.47 *Leanness* E: 21.4 ± 3.45 R: 21.1 ± 3.68 NC: 22.2 ± 4.18 *Non-Leanness* E: 21.9 ± 3.55 R: 22.3 ± 3.01 NC: 21.9 ± 3.00	BMI (self-reported data) *Leanness sports* E: 20.7 ± 2.02 R: 21.4 ± 2.47 NC: 21.5 ±1.90 *Non-Leanness sports* E: 22.2 ± 1.82 R: 21.7 ± 2.19 NC: 22.2 ± 2.65 Current−Ideal W (kg) *Leanness sports* E: 4.50 ± 2.72 R: 3.70 ± 3.03 NC: 2.44 ± 2.61 *Non-Leanness sports* E: 3.10 ± 3.07 R: 2.93 ± 3.46 NC: 2.54 ± 2.89	According to Stunkard et al. [29] CBI; IBI; sport-IBI *Leanness sports* E: 3.54 ± 1.01; 2.20 ± 0.86; 2.71 ± 0.83 R: 4.22 ± 1.07; 3.07 ± 0.87; 2.90 ± 0.82 NC: 4.25 ± 0.98; 3.02 ± 0.75; 2.94 ± 0.73 *Non-Leanness sports* E: 3.94 ± 1.14; 3.00 ± 0.73; 3.43 ± 0.75 R: 4.13 ± 1.06; 3.02 ± 0.67; 3.40 ± 0.79 NC: 4.20 ± 0.89; 3.02 ± 0.75; 3.38 ± 0.86 BID; Sport-BID *Leanness sports* E: −1.34 ± 1.09; −0.83 ± 1.22 R: −1.15 ± 1.32; −1.32 ± 1.29 NC: −1.23 ± 1.09; −1.31 ± 0.99 *Non-Leanness sports* E: −0.94 ± 0.93; −0.48 ± 1.17 R: −1.11 ± 0.87; −0.74 ± 1.06 NC: −1.00 ± 0.83; −0.82 ± 1.03	Athletes in sports focused on leanness had higher levels of body dissatisfaction, regardless of the level of participation. Greater levels of body dissatisfaction were reported by elite athletes regardless of sport type, while no differences were found between amateur and non-competitive athletes. More than 60% of elite athletes in lean-focused and non-focused sports indicated pressure from coaches regarding body shape	10 moderate
Krentz and Warschburger (2013) [41] -1-year longitudinal study-	Adolescents practicing aesthetic sports from 6 elite sports schools and Olympic training centers in Germany were selected and measured 2 times one year apart. Time 1 *Exercise (h/w)* *Total:* 13.9 ± 6.6 *F:* 12.1 ± 5.6 *M:* 16.5 ± 7.2 *Competition in the specific sport (yrs)* *Total:* 6.9 ± 2.3 *F:* 6.7 ± 2.6 *M:* 7.1 ± 1.7	Sixty-five adolescents: 27 boys and 38 girls practicing the following sports: gymnastics (12 M and 8 F), ice figure skating (4 M and 15 F), diving (7 M and 3 F), ballet (3 M and 4 F), roller-skate figure skating (1 M and 5 F), rhythmic gymnastics (3 F)	Time 1 Total:14.0 ± 2.3 F:14.0 ± 2.4 M: 14.1 ± 2.1	BMI (self-reported data) Time 1 Total: 18.1 ± 2.4 F: 18.1 ± 2.6 M: 18.2 ± 2.0	According to Thompson and Gray [37] Sport-BID Time 1 Total: −0.5 ± 1.0 F: −0.8 ± 1.0 M: 0.0 ± 0.7 Time 2 Total: −0.6 ± 1.1 F: −0.9 ± 1.1 M: −0.2 ± 1.1 Correlations between BID at times 1 and 2 were significant	Rather high stability of sports correlates was observed over one year with a significant increase in the social pressure of the sports environment among girls but not among boys. This may be due to the increasing importance of appearance for girls during adolescence not only outside the world of sports but also particularly in aesthetic sports. The study shows that sports-related BID is not predictive of disordered eating when other sport-related variables are also included	11 moderate
Pinto et al. (2019) [42] -cross-sectional-	Elite male artistic gymnasts, from three training centers in the state of Sao Paulo, Brazil, all of whom were in full-time training at the time of the study International level: 7 (3 Olympians); national level: 10 Training: 6 days/week, for a median of 6 h/day Median experience as gymnast of 14 years	70 male athletes	22.5 ± 3.2	BMI (self-reported data) 23.6 ± 1.9	According to Frederick et al. [43,44] CBI: 5.2 ± 1.2 Healthy: 5.0 ± 1.3 IBI: 6.2 ± 0.8 BID: 1.13 ± 1.09 (range: −2; 3) 87.5% of athletes desired a larger/stronger body shape than their self-reported current	BI perceptions and attitudes toward the influence of body weight on performance differed greatly. Some athletes had the desire to lose weight thinking about its positive impact on their performance, while others felt that weight was unimportant and they were focused on feeling vigorous and vital	5 low
Santarnecchi and Dettore (2012) [45] -cross-sectional-	Italian male competitive and non-competitive bodybuilders	180 subjects divided into 3 groups:60 competitive bodybuilders (CB), 60 noncompetitive bodybuilders (NCB), 60 non-training subjects (NT)	CB: 33 ± 7 NCB: 32 ± 10 NT: 33 ± 8	BMI CB: 27.93 NCB: 24.60 NT: 25.02 Significant correlations: -BMI-current body type Fat -BMI-self-reported %F -BMI-current body type Muscle Mass	According to Body Building Image Grid-Scaled (BIG S) of Hildebrandt et al. [33] *Current body type–Fat* CB: 27.33 ± 17.84 NCB: 41.67 ± 18.33 NT: 50.67 ± 18.40 *Current body type–Muscle Mass* CB: 64.33 ± 12.12 NCB: 46.83 ± 18.55 NT: 29.33 ± 15.17 *Ideal body type–Fat* CB: 14.33 ± 9.63 NCB: 30.50 ± 17.02 NT: 37.33 ± 16.04 *Ideal body type–Muscle Mass* CB: 75.17 ± 16.00 NCB: 53.17 ± 9.83 NT: 42.00 ± 16.95	The study showed an almost linear trend of increasing current and ideal body fat levels and decreasing muscle mass levels in the transition from competitive bodybuilders to non-training subjects	7 low
Torres-McGehee et al. (2012) [46] -cross-sectional-	American cheerleaders	136 Female collegiate cheerleaders According to position: 54 bases, 61 flyers, and 21 back spots. According to academic status 48 freshmen, 42 sophomores, 21 juniors, 25 seniors	20.4 ± 1.3	(self-reported data) H: 160.2 ± 8.1 cm W: 57.2 ± 8.3 kg BMI: 22.3 ± 2.8	According to Stunkard et al. [29] modified by Bulik et al. [47] IBI = 3.4 ± 4.3	Cheerleaders, especially flyers, appear to be at risk for eating disorders, with the greatest BID when wearing their most revealing uniforms (i.e., midriffs). Universities, colleges, and the national governing bodies of these squads need to focus on preventing eating disorders and BID and promoting self-esteem	13 high
Voelker et al. (2014) [48] -cross-sectional-	American figure skaters across five US states	272 female figure skaters with 9.48 ± 4.15 years of skating experience. Of them, 83 elite (31%) with national/international competitions	15.63 ± 3.02	BMI: 20.79 ± 3.47	According to Thompson and Gray [37] BID: −0.85 ± 1.23 Sport-BID: −1.00 ± 1.48	Weight and appearance concerns, body dissatisfaction both general and sport-related, and positive perfectionism may be relevant in detecting disordered eating in female skaters	14 high
Voelker et al. (2017) [49] -cross-sectional-	American figure skaters across five US states	29 male figure skaters (23 with national/international competition) Years skating 11.26 ± 5.47	18.45 ± 4.15	BMI: 22.53 ± 3.94	According to Thompson and Gray [37] Sport-BID: −0.26 ± 0.77	Body mass index, sport-related weight pressures, and sport-related body dissatisfaction explained 30% of the variance in eating disorder symptomatology	9 moderate

Note: F: females; M: males; BMI: body mass index; H: height; W: weight; UW: underweight; NW: normal weight; OW: overweight; CBI: current body image; IBI: ideal body image; Sport-IBI = ideal body image for the sport practiced; BID = body image dissatisfaction (=ideal body image—current body image); Sport-BID = body image dissatisfaction in sport (=sport-IBI—current body image).

**Table 2:** BID by gender: results of the meta-analysis.

	**Females**			**Males**			**%**		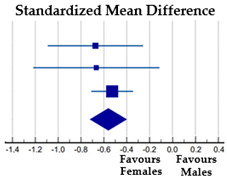
**Subgroup**	**N**	**Mean**	**SD**	**N**	**Mean**	**SD**	**Weight**	**SMD [95% CI]**	
Elite aesthetic athletes [36]	101	−0.88	1.21	30	−0.11	0.83	14.86	−0.67 [−1.09, −0.26]	
Non-elite aesthetic athletes [36]	99	−0.51	1.19	15	0.27	0.96	8.51	−0.67 [−1.22, −0.14]	
Non-aesthetic athletes [36]	253	−0.81	1.31	227	−0.17	1.09	76.64	−0.53 [−0.71, −0.35]	
Total (random effects)	453			272			100.00	−0.56 [−0.72, −0.40]	

Note: Squares represent the individual studies and vary in size according to the weights assigned to each; diamond summaries all individual studies combined together and averaged: its location represents the estimated effect size and the width reflects the precision of the estimate.

**Table 3:** BID by type of sports in female athletes.

	**Aesthetic Sports**			**Non-Aesthetic Sports**			**%**		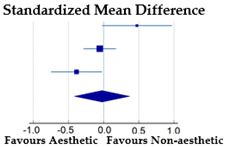
**Study**	**N**	**Mean**	**SD**	**N**	**Mean**	**SD**	**Weight**	**SMD [95% CI]**	
Borrione et al. [28]	20	−0.5	0.8	80	−1.0	1.1	26.91	0.47 [−0.02, 0.97]	
Francisco et al. [36]	101	−0.88	1.21	253	−0.81	1.31	39.76	−0.05 [−0.29, 0.18]	
Kong and Harris [40]	80	−1.34	1.09	48	−0.94	0.93	33.33	−0.39 [−0.75, −0.23]	
Total (random effects)	201			381			100.00	−0.02 [−0.42, 0.37]	

Note: Type of sports considered: Borrione et al.: aesthetic sports—rhythmic gymnastics; non-aesthetic sports—basketball, volleyball, Taekwondo, fitness; Francisco et al.: aesthetic sports—gymnastics and dance; non-aesthetic sports—not reported; Kong and Harris: aesthetic sports—leanness-focused sports (dance, performance sports (gymnastics, cheerleading), cycling/endurance sports, long distance running, lightweight boxing, and lightweight rowing); non-aesthetic sports—non-leanness-focused sports (ball sports such as football, netball, soccer, bat/stick sports such as hockey, cricket, baseball, racquet sports, water polo, and heavyweight rowing). Squares represent the individual studies and vary in size according to the weights assigned to each; diamond summaries all individual studies combined together and averaged: its location represents the estimated effect size and the width reflects the precision of the estimate.

**Table 4:** BID by the level of sports in female athletes.

	**Elite Level**			**Non-Elite Level**			**%**		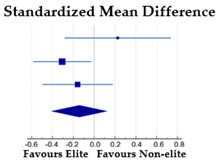
**Study**	**N**	**Mean**	**SD**	**N**	**Mean**	**SD**	**Weight**	**SMD [95% CI]**	
Borrione et al. [28]	20	−0.5	0.8	61	−0.7	0.9	20.93	0.23 [−0.28, 0.74]	
Francisco et al. [36]	101	−0.88	1.21	99	−0.51	1.19	43.39	−0.31 [−0.59, −0.03]	
Kong and Harris. [40]	80	−1.34	1.09	59	−1.15	1.32	35.68	−0.16 [−0.50, 0.18]	
Total (random effects)	201			219			100.00	−0.14 [−0.41, 0.12]	

Note: Squares represent the individual studies and vary in size according to the weights assigned to each; diamond summaries all individual studies combined together and averaged: its location represents the estimated effect size and the width reflects the precision of the estimate.

**Table 5:** BID by weight status in female athletes.

	**Underweight**			**Normal-Weight**					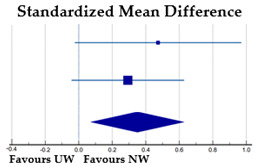
**Subgroup**	**N**	**Mean**	**SD**	**N**	**Mean**	**SD**	**Weight**	**SMD [95% CI]**	
UW (international RG) vs. NW (controls) [28]	20	−0.5	0.8	80	−1.0	1.1	31.55	0.47 [−0.02, 0.97]	
UW (national RG) vs NW (controls) [28]	61	−0.8	0.9	80	−1.0	1.1	68.45	0.29 [−0.04, 0.63]	
Total (random effects)	81			160			100.00	0.35 [0.07, 0.63]	

Note: UW = underweight; RG = rhythmic gymnasts; NW = normal weight. Squares represent the individual studies and vary in size according to the weights assigned to each; diamond summaries all individual studies combined together and averaged: its location represents the estimated effect size and the width reflects the precision of the estimate.

**Table 6:** Effect of gender on Sport-BID in elite aesthetic athletes.

	**Females**			**Males**					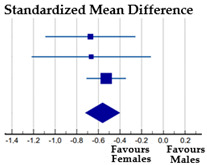
**Subgroup**	**N**	**Mean**	**SD**	**N**	**Mean**	**SD**	**Weight%**	**SMD [95% CI]**	
Elite dancers [23]	53	−1.45	1.32	13	0.08	0.95	17.05	−1.20 [−1.85, −0.56]	
Elite gymnasts [23]	50	−1.20	1.04	19	−0.58	1.02	22.66	−0.59 [−1.13, −0.05]	
Elite figure skaters [48,49]	272	−1.00	1.48	29	−0.26	0.77	36.18	−0.52 [−0.90, −0.13]	
Elite aesthetic sports [41]	38	−0.80	1.00	27	0.00	0.70	24.10	−0.89 [−1.41, −0.37]	
Total (random effects)	413			88			100.00	−0.74 [−1.03, −0.46]	

Note: Squares represent the individual studies and vary in size according to the weights assigned to each; diamond summaries all individual studies combined together and averaged: its location represents the estimated effect size and the width reflects the precision of the estimate.

## Data Availability

Not applicable.

## Supplementary Materials

The following supporting information can be downloaded at: https://www.mdpi.com/article/10.3390/ijerph20065228/s1: Table S1: Prisma 2020 Checklist; Table S2: Assessment of risk of bias of included studies by NOS (score: 0–16, with higher scores indicating lower risk of bias). Each item is followed by its range. Reference [79] is cited in the supplementary materials.
