# Supplementary material for: The Impact of Sports Involvement on Body Image Perception and Ideals: A Systematic Review and Meta-Analysis
Source: Int J Environ Res Public Health. 2023 Mar 22;20(6):5228. doi: 10.3390/ijerph20065228 (PMC10049477; doi:10.3390/ijerph20065228)
Supplement: Supplementary file 1 [file ijerph-20-05228-s001.zip › ijerph-2280098.Table S2 NOS.pdf]

**Table S2.** Assessment of risk of bias of included studies by NOS (score: 0-16, with higher scores indicating low risk of bias). Each item is followed by its range.

| Reference | Clarity of Stated aim (0-2) | Sample Rappresent-ativeness (0-2) | Sample Size (0-2) | Non-Respondents (0-2)* | Ascertainment of the Exposure (0-2) | Control of Confounding factors (0-1) | Comparability (0-1) | Assessment of the Outcome (0-2) | Statistical Tests (0-2) | NOS Score |
|---|---|---|---|---|---|---|---|---|---|---|
| Borrione et al. (2013) [28] | 2 | 1 | 1 | 0 | 2 | 1 | 1 | 1 | 2 | 11 |
| Cardoso et al. (2021) [30] | 2 | 2 | 1 | 0 | 2 | 1 | 1 | 1 | 2 | 12 |
| Da Silva et al. (2016) [31] | 2 | 0 | 0 | 0 | 2 | 0 | 1 | 1 | 0 | 6 |
| Devrim et al. (2018) [32] | 2 | 1 | 1 | 0 | 2 | 1 | 1 | 1 | 1 | 10 |
| de Medeiros Eufrásio et al. (2021) [34] | 2 | 1 | 2 | 0 | 2 | 0 | 1 | 1 | 2 | 11 |
| Francisco et al. (2012) [23] | 2 | 1 | 1 | 0 | 2 | 1 | 1 | 2 | 2 | 12 |
| Francisco et al. (2013) [36] | 2 | 1 | 1 | 0 | 2 | 1 | 1 | 2 | 2 | 12 |
| Godoy-Izquierdo and Díaz (2021) [38] | 2 | 1 | 1 | 0 | 2 | 1 | 0 | 1 | 2 | 10 |
| Kong and Harris (2015) [40] | 2 | 2 | 2 | 0 | 2 | 0 | 1 | 1 | 0 | 10 |
| Krentz and Warschburger (2013) [41] | 2 | 1 | 1 | 2 | 2 | 0 | 1 | 1 | 1 | 11 |
| Pinto et al. (2019) [42] | 2 | 0 | 0 | 0 | 2 | 0 | 0 | 1 | 0 | 5 |
| Santarnecchi and Dettore (2012) [45] | 2 | 0 | 0 | 0 | 2 | 0 | 1 | 1 | 1 | 7 |
| Torres-McGehee et al. (2012) [46] | 2 | 1 | 2 | 1 | 2 | 1 | 1 | 1 | 2 | 13 |

| | | | | | | | | | | |
|---|---|---|---|---|---|---|---|---|---|---|
| Voelker et al. (2014) [48] | 2 | 2 | 2 | 1 | 2 | 1 | 1 | 1 | 2 | 14 |
| Voelker et al. (2017) [49] | 2 | 1 | 1 | 0 | 2 | 0 | 0 | 1 | 2 | 9 |

*As carried out previously [55], we assigned the score 0 to “iii. One or none of three” in the Non-respondents category of the NOS adapted scale [27].
